# Supplementary material for: Life-time experience of violence among women and girls living with disability in Nepal
Source: PLoS One. 2025 Jun 25;20(6):e0326659. doi: 10.1371/journal.pone.0326659 (PMC12193803; doi:10.1371/journal.pone.0326659)
Supplement: Attachment — Submitted filename: Comment.pdf [file pone.0326659.s001.pdf]

I greatly appreciate the authors for addressing the issue of violence against women with disabilities—a topic that is often overlooked and given low priority in our context. This study provides valuable insights into the prevalence and nature of such violence. While the paper is well-organized and adheres to journal standards, I have some observations and suggestions for improvement to make it more comprehensive.

Abstract

- While the abstract mentions that half of women with disabilities have experienced violence, it would be more insightful to specify which age groups are most affected and the types of violence they face, such as sexual, physical, or others, rather than focusing solely on lower odds. Additionally, there is a lack of coherence between the abstract and recommendations. The abstract emphasizes lower odds, while the recommendations highlight a high prevalence of violence and propose policies incorporating intersectionality. This inconsistency could be addressed to improve clarity and alignment.

Background

- Referencing within the background section is inconsistent. For example, the sixth line references a United Nations report within the text, whereas other sections adopt the Vancouver style.
- Moreover, I recommend adding background information on the specific types of violence faced by women with disabilities—such as sexual, physical, or domestic violence, globally, regionally or if possible from Nepal to underline the significance of the study and its policy implications. Also, a brief discussion category of the Government of Nepal for disability, and the category that study has consider so that reader can understand why some of the disability is not consider or vice versa.

Methods

- The categorization of disabilities could be improved by aligning it with the Nepalese government's guidelines, which classify disabilities into ten types. The authors should describe this framework in both the background and methods sections to provide clarity. Including a table with operational definitions for the types of violence and disabilities would also enhance the paper's comprehensiveness. Additionally, the term "Brahman" should be used consistently instead of "Braman" to align with the terminology in Nepal’s national population and housing census.

Results

- Figures are missing from the results section, which makes it difficult to provide specific feedback. However, I suggest using the term "vocal and speech-related disability" rather than "speech/voice disability" for standard terminology.
  It is notable that violence is more prevalent among younger women with disabilities compared to older ones in this study which is commonly seen as well. Therefore the discussion should explore the types of violence younger women are more susceptible to, such as sexual violence or neglect (e.g., menstrual hygiene), as this specificity would provide valuable insights.
- While education status is not statistically significant in relation to violence, the fact that violence is slightly higher among educated women is surprising. A potential explanation could be their increased social engagement, which may expose them to stigma or bullying by peers and teachers. Discussing such non-significant in number yet intriguing findings would enrich the paper.

Discussion

- The discussion section is well presented. However, I recommend incorporating an analysis of the types of violence experienced by specific age groups and discuss it more and according to provinces too. This would enable Nepal to develop targeted, evidence-based policies that are contextualized to local realities.

Conclusion

- The conclusion feels overly generic. While the results highlight factors associated with violence, the conclusion should provide more specific recommendations. For example, critical issues like denial of services should be addressed explicitly, alongside suggestions for law reform. Concluding with a focus on the significance of further research and its potential impact would make the conclusion more impactful and comprehensive.
