# Supplementary material for: Life-time experience of violence among women and girls living with disability in Nepal
Source: PLoS One. 2025 Jun 25;20(6):e0326659. doi: 10.1371/journal.pone.0326659 (PMC12193803; doi:10.1371/journal.pone.0326659)
Supplement: Attachment — Submitted filename: Tracked changed_ Reviewed_AE.docx [file pone.0326659.s002.docx]

**Life-time experience of violence among women and girls living with disability in Nepal**

Padam Simkhada,^1^ Sapana Basnet^2^ Shaurabh Sharma^3^ Edwin van Teijlingen, PhD,^4^ Sharada Prasad Wasti,^5^ Tikadevi Dahal,^6^ Joshua Okyere^1,7^* Ram Chandra Silwal^8^ and Manita Pyakurel^8^

1. School of Human and Health Sciences, University of Huddersfield, West Yorkshire, United Kingdom
2. Sightsavers International, West Sussex, United Kingdom
3. Humanity & Inclusion – Handicap International, Addis Ababa, Ethiopia
4. Centre for Midwifery & Women’s Health, Bournemouth University, Dorset, United Kingdom
5. School of Human Sciences, University of Greenwich, London, United Kingdom
6. Nepal Disabled Women Association (NDWA), Kathmandu, Nepal
7. Department of Population and Health, University of Cape Coast, Cape Coast, Ghana.
8. Green Tara Nepal, GPO, CPC, Kathmandu, Nepal

***Corresponding author:** Joshua Okyere

Email: [joshuaokyere54@gmail.com](mailto:joshuaokyere54@gmail.com)/joshua.okyere@hud.ac.uk  ORCID: [0000-0003-4080-7522](https://eur02.safelinks.protection.outlook.com/?url=https%3A%2F%2Forcid.org%2F0000-0003-4080-7522%3Flang%3Den&data=05%7C02%7CP.P.Simkhada%40hud.ac.uk%7C5d82ff3af2324021f4fc08dc2043cb22%7Cb52e9fda06914585bdfc5ccae1ce1890%7C0%7C0%7C638420723620676220%7CUnknown%7CTWFpbGZsb3d8eyJWIjoiMC4wLjAwMDAiLCJQIjoiV2luMzIiLCJBTiI6Ik1haWwiLCJXVCI6Mn0%3D%7C0%7C%7C%7C&sdata=CEYKBRm9RihJReAZXjrIClpEozgVzoVz6wyShE70pQE%3D&reserved=0)

**Abstract**

**Background**: Violence against women and girls with disabilities remains a serious, yet underexplored, global concern. This paper aims to investigate the prevalence and factors associated with life-time experience of violence within this vulnerable population in Nepal.

**Methods**: This cross-sectional study was conducted in 28 municipalities representing all seven provinces as well as all three ecological regions of Nepal. Participants were girls and women with disabilities (aged 15-59 years). A total of 1,294 questionnaires were completed in 2021 using the KoBo toolbox application on a smartphone or tablet. Cross-tabulations and chi-square tests were performed in STATA 18. Also, bivariable and multivariable logistic regression models were fitted to establish associations.

**Results**: Overall, 457 (35.32%) women living with disabilities had ever experienced violence at a point in their lifetime. Psychological/emotional violence was the most prevalent violence (74.40%) followed by physical violence (31.07%) and denial of services (28.67%). Age was positively associated with the likelihood of experiencing violence. Women belonging to the Brahman/Chhetri ethnic group had reduced odds of violence [AOR=0.56; 95%CI: 0.37–0.85] compared to Hill Dalits. Divorced or separated women showed a markedly higher likelihood of experiencing violence [AOR=6.69; 95%CI: 2.31–19.40] compared to currently married women. Participants who had not witnessed violence against other women exhibited significantly lower odds of experiencing violence [AOR=1.86; 95%CI: 1.20–2.89]. Women living in provinces other than Province 1 reported significantly reduced odds of experiencing violence, with the lowest odds observed in Sudurpaschim [AOR=0.04; 95%CI: 0.02–0.09] and Lumbini [AOR=0.08; 95%CI: 0.05–0.15].

**Conclusion**: In conclusion, the experience of violence varies significantly by disability type. There is a need for comprehensive, disability-inclusive policies that address the specific risks identified. Policy interventions in Nepal must focus on improving access to services for women with vocal and speech-related disabilities, ensuring that healthcare and support services are inclusive of alternative communication methods. Additionally, tailored support for divorced or separated women and age-appropriate interventions for young girls with disabilities are crucial in mitigating their increased vulnerability to violence.

**Keywords**: Violence Against Women; Girls with Disability; Cross-sectional Survey, South Asia; Nepal

**Background**

Violence against women and girls is a serious global health problem as highlighted by the World Health Organization [1]. It is a problem disproportionately affecting women living with disabilities, although exact data on this population are often not available in low-income countries, such as Nepal. Globally, one in five people, nearly 1.3 billion live with disability (PWDs), and most (80%) reside in the global south, and it is estimated that some three-quarters are female [2]. In Nepal, 2.2 percent of the total population live with at least one type of disability [3]; half of this the population living with disabilities are females. To bring the issue of disability into lime light, the Nepalese government in 2017 passed the Act on the Rights of Persons with Disabilities which categorizes disabilities into ten types based on the impairment (i.e., physical, vision related, hearing, deaf-blind, mental/psychological, intellectual disability, hemophilia, autism, and multiple disabilities), and also into four types based on the degree of severity [4].

Several studies and official reports indicate that PWDs are often one of the most disadvantaged populations in society [1,5-7]. A recent review identified several important gaps in research around violence, such as lack of sex and disability disaggregation [8]. Violence against women and girls living with disabilities has adverse effects on their wellbeing, leading to a higher risk of severe distress, anxiety, and depression [9], and poorer self-reported health outcomes [10]. As such, it is imperative to understand the factors that exacerbates women with disabilities’ risk of becoming victims of violence.

The 2021 Nepal Census records 654,782 PWDs in the country [10], and nearly half (45.8%) of them are females. In Nepal, the situation is exacerbated due to the caste system, in which individuals are stratified into disparate positions from birth by social and power structures [11,12]. A review by Thapa and colleagues [12] highlighted that “Caste-based inequity impacts upon all aspects of an individual’s well-being including violence and everyday life risks.” Low-caste women and girls living with disabilities are more likely to experience gender-based violence (GBV) and discrimination [11]. To combat the problem Nepal established a Gender-Based Violence and Gender Equality Funds and adopted a gender-equality-and-development frame to facilitate the elimination of all forms of violence, including violence against PWDs [13,14]. There was also the implementation of the One-Stop Crisis Management Centers (OCMC) to provide a sanctuary for victims of violence [15].

In spite of the implemented policies, it is crucial to note that prior studies conducted in Nepal have revealed a diverse prevalence of lifetime experiences of violence against women and girls with disabilities. Notably, Puri et al. [11] identified an overall lifetime prevalence of 57.7 percent across three districts, while Gupta et al. [16] reported 25.3 percent violence among married women in three districts of the Terai region. However, a significant drawback of these studies lies in their limited geographical focus, which does not offer a comprehensive understanding of the national level. Consequently, there is a pressing need for research that encompasses all provinces in Nepal, to provide a nationally representative perspective on this critical issue. This paper aims to investigate the prevalence and factors associated with life-time experience of violence within this vulnerable population in Nepal.

**Methods**

***Study population and design***

The study follows a quantitative research approach and specifically adopts a cross-sectional design. We conducted quantitative surveys in 14 districts representing all seven of Nepal’s provinces (Province 1, Province 2, Bagmati, Gandaki, Lumbini, Karnali, and Sudurpaschim). The study spanned districts in the three ecological areas. In the mountains area, we worked in Jumla. Also, the following districts were in the Hills ecological area; Dhankuta, Kavre, Kathmandu, Gorkha, Kaski, Argakhachi, Surkhet, and Achham. For the Terai ecological area, the following districts were covered: Morang, Siraha, Dhanusha, Dang, and Kanchanpur. A total of 1,294 women and girls with disability aged 15 to 59 years participated in this study. However, women and girls with mental health problems or with severe intellectual disabilities were excluded as the research team did not have the capacity (i.e. trained interviewers in mental health) to survey women and girls with specialized needs.

***Sampling procedure***

A multistage sampling technique [17] was applied, first, all districts were stratified based on (a) the three geographical regions and (b) seven provinces, next 14 districts were selected from these strata using a lottery method. In the second stage, 28 sites (16 municipalities, 9 rural municipalities, and 3 metropolitan cities) were chosen. Finally, a list of women and girls with disability for the selected municipalities was prepared with the help of members from the women and children development section of the municipality office. A representative from the Nepal Disabled Women Association (NDWA) and 18 female data enumerators collaboratively identified disabled women and girls' households for each area. As a result, 45 to 46 girls and women with disabilities were recruited from each rural municipality, urban municipality and metropolitan city. Participants had to have been residents of the study areas for at least 12 months preceding the survey.

It must be noted that this study is part of a larger project in Nepal to reduce violence against women with disabilities. The sample size was informed by Puri et al. [11] study that found a 57.7% violence prevalence among women with disabilities. In our project, we estimated that were would be a 5% reduction during the project interventions. As such, the following parameters were considered in the sample size estimation: significance level=0.05; power=0.8; p1 = 0.577; p2 = 0.527 (5% decreased at end line); n2/n1(ratio) = 1; current mean cluster size = 4; intra-cluster correlation coefficient = 0.2; maximum expected cluster size = 5; minimum expected cluster size = 3; design effect: unadjusted = 1.6; and design effect: adjusted = 1.6125. This resulted in an adjusted sample size of 1,294.

**Study variables**

Life-time experience of violence was the outcome variable. Life-time is this context means that we are not restricted to the past 12 months or 1 month preceding the survey. Rather, we were interested in whether women living with disability had ever experienced any kind of violence at any point in their life time. This was derived from the question, “Have you ever experienced any form of violence?” The response categories were yes and no. Responding yes meant that the individual had ever experienced violence in their lifetime. Additionally, the specific violence type (i.e., physical violence, sexual violence, forced/child marriage, psychosocial abuse, and spousal violence) that has ever been experienced was also covered.

Based on literature reviewed [8,11,16], nine potential explanatory variables we selected, including age (i.e., 15 to 19; 20 to 24; 25 to 29; 30 to 34; 35 to 39; and 40 years and over), ethnicity (i.e., Hill Dalit, Terai Dalit, Hill Janajati, Terai Janajati, Brahman/Chhetri), religion (i.e., Hindu, Buddhist, Muslim, Kirat and Christian), ever attended school, marital status (i.e., currently married, never married, divorced/separated, and widowed), age at first marriage (i.e., before 20 years, at 20 year or older), whether the person received disability allowance, knew policies and laws that support victims of violence, and ever witnessed violence against women.

***Data collection procedures***

Study questionnaires were prepared and adopted from the Nepal Demographic and Health Survey, 2016 (NDHS, 2017), and Nepal Multiple Indicator Cluster Surveys 2014 (CBS, 2015) whose tools had already been used in Nepal. A pilot study [18] examined the adequacy of the questions, clarity/wording of questions, adequacy of possible responses (pre-coded), sequence/flow, and appropriate administration techniques. Based on the feedback from the pre-testing, all tools were finalized and used for the final data collection. Survey data were collected by trained enumerators using the KoBo application [19] on smartphones or tablets. Every day data was sent to a web server by the data enumerators and saved by the researcher. Data was collected between August 30, 2021 to October 29, 2021.

***Statistical analyses***

The data keyed into KoBo, the computer-assisted personal interviewer, was extracted from the server and exported to STATA version 18 (StataCorp, College Station, TX, USA) for data management and analyses. The data were encoded to ensure that all numeric data were in their right format. Following that, a descriptive analysis was performed to determine the distribution of the sample and variables of interest. Pearson’s chi-square test was then computed to determine whether there were significant differences in the estimated prevalence of life-time experience of violence across the explanatory variables. As the outcome variable is binary in nature, binary logistic regression was fitted. First, the bivariable analysis was computed to ascertain the association between each explanatory variable and the likelihood of experiencing violence. The results were presented in odds ratio at a 95% confidence interval. Following that, a multivariable logistic regression model was fitted to adjust for the effect of all the explanatory variables. This was represented by an adjusted odds ratio at a 95% confidence interval.

**Ethical considerations**

Ethical approval was provided by the Nepal Health Research Council (NHRC) (registration number 339/2020P). Both written and oral informed consent was sought from all participants. Each participant was assured of utmost confidentiality and privacy. We assured them that the data collected will be anonymised to protect their identities.

**Results**

**Characteristics of study participants**

Table 1 shows the sample distribution. Most participants were aged 40 years and above (27.43%). In terms of ethnicity, the majority are Brahman/Chhetri (42.50%). Most participants identify as Hindu (88.25%), had not received any formal education (51.78%), and were never married (50.39%). However, a majority of the sample reported being married before the age of 20 years (82.61%). Most participants received a disability allowance (54.33%) and had ever witnessed violence against women (91.04%). Provincial distribution shows the highest representation from Gandaki Province (15.07%).

**Table 1: Distribution of sample and experience of life-time prevalence of violence**

| **Characteristics** | **Sample**  **n (%)** | **Proportion ever experienced violence**  **n(%)** | **Chi-square value**  χ***^2^*; p-value** |
| --- | --- | --- | --- |
| **Overall** | **1294 (100)** | **457 (35.32)** |  |
| **Age** |  |  | χ***^2^*=24.4615; p<0.001** |
| 15-19 years | 184 (14.22) | 39 (21.20) |  |
| 20-24 years | 212 (16.38) | 75 (35.38) |  |
| 25-29 years | 242 (18.70) | 95 (39.26) |  |
| 30-34 years | 163 (12.60) | 71 (43.56) |  |
| 35-39 years | 138 (10.66) | 56 (40.58) |  |
| 40 years and above | 355 (27.43) | 121 (34.08) |  |
| **Ethnicity** |  |  | χ***^2^*=27.1811; p<0.001** |
| Hill Dalit | 190 (14.68) | 58 (30.53) |  |
| Terai Dalit | 61 (4.71) | 30 (49.18) |  |
| Hill Janajati | 224 (17.31) | 95 (42.41) |  |
| Terai Janajati | 269 (20.79) | 114 (42.38) |  |
| Brahman/Chhetri | 550 (42.50) | 160 (29.09) |  |
| **Religion** |  |  | χ***^2^*=35.3166; p<0.001** |
| Hindu | 1142 (88.25) | 374 (32.75) |  |
| Buddhist | 47 (3.63) | 28 (59.57) |  |
| Muslim | 33 (2.55) | 18 (54.55) |  |
| Kirat | 12 (0.93) | 10 (83.33) |  |
| Christian | 60 (4.64) | 27 (45.00) |  |
| **Education** |  |  | χ***^2^*=**0.0766; p= 0.782 |
| No | 670 (51.78) | 239 (35.67) |  |
| Yes | 624 (48.22) | 218 (34.94) |  |
| **Marital status** |  |  | χ***^2^*=39.0610; p<0.001** |
| Currently married | 550 (42.50) | 202 (36.73) |  |
| Never married | 652 (50.39) | 206 (31.60) |  |
| Divorced/separated | 35 (2.70) | 29 (82.86) |  |
| Widowed | 57 (4.40) | 20 (35.09) |  |
| **Age at first marriage** |  |  | χ***^2^*=8.9892; p= 0.003** |
| Before 20 years | 1069 (82.61) | 385 (33.49) |  |
| At 20 year or older | 225 (17.39) | 99 (44.00) |  |
| **Receive disability allowance** |  |  | χ***^2^*=**1.037; p= 0.308 |
| No | 591 (45.67) | 200 (33.84) |  |
| Yes | 703 (54.33) | 257 (36.56) |  |
| **Knowledge on policies and laws that support victims of violence** |  |  | χ***^2^*=**2.4869; p= 0.115 |
| Yes | 269 (20.79) | 84 (31.23) |  |
| No | 1025 (100) | 373 (36.39) |  |
| **Ever witnessed violence against women** |  |  | χ***^2^*=34.9402; p<0.001** |
| Yes | 1178 (91.04) | 387 (32.85) |  |
| No | 116 (8.96) | 70 (60.34) |  |
| **Provinces** |  |  | χ***^2^*=225.3497; p<0.001** |
| Province 1 | 185 (14.30) | 125 (67.57) |  |
| Province 2 | 182 (14.06) | 96 (52.75) |  |
| Bagmati | 187 (14.45) | 90 (48.13) |  |
| Gandaki | 195 (15.07) | 51 (26.15) |  |
| Lumbini | 187 (14.45) | 26 (13.90) |  |
| Karnali | 190 (14.68) | 56 (29.47) |  |
| Sudurpaschim | 168 (12.98) | 13 (7.74) |  |

**Life-time prevalence of violence among women and girls living with disabilities**

Overall, 457 (35.32%) women living with disabilities had ever experienced violence at a point in their lifetime (Table 1). Significantly high prevalence of violence was found among those age 30–34 years (43.56%), Terai Dalit participants (49.18%), and the Kirat group (83.33%). Divorced or separated individuals reported the highest prevalence of violence (82.86%). Persons who married at age 20 or older (44.00%) and those who lacked knowledge of policies on violence (36.39%) reported a high prevalence of violence. Interestingly, those who have not witnessed violence against women reported a substantially higher prevalence of personal violence (60.34%) compared to those who had. Provincially, participants from Province 1 had the highest prevalence of violence (67.57%).

**Types of violence experienced by women and girls living with disability**

The results show that psychological/emotional violence was the most prevalent violence (74.40%) followed by physical violence (31.07%) and denial of services (28.67%). Sexual violence (2.19%) and forced/child marriage (2.63%) were the least prevalent type of violence among women living with disabilities (Table 2).

The highest prevalence of sexual violence was observed among women in Lumbini province (19.23%). A higher prevalence of sexual violence was observed among women aged 15-19 years (7.69%) and Muslims (5.56%). Women in the Karnali province (51.79%), Terai Dalit women (40.00%), those aged 30-34 years (38.03%), and those who professed Buddhism (39.29%) reported a higher prevalence of physical violence. Hill Janajati women reported a higher prevalence of psychological (78.24%), spousal (20.69%), and sexual violence (4.21%). Interestingly, participants with formal education reported high prevalence of all the types of violence.

Table 2: Distribution of the prevalence of the types of violence among women living with disability

| **Characteristics** | **Sexual violence**  **n (%)** | **Physical violence**  **n (%)** | **Force/child marriage**  **n (%)** | **Denial of services**  **n (%)** | **Spousal violence**  **n (%)** | **Psychological violence**  **n (%)** |
| --- | --- | --- | --- | --- | --- | --- |
| **Overall** | **10 (2.19)** | **142 (31.07)** | **12 (2.63)** | **131 (28.67)** | **61 (13.35)** | **340 (74.40)** |
| **Age** |  |  |  |  |  |  |
| 15-19 years | 3 (7.69) | 12 (30.77) | 1 (2.56) | 10 (25.64) | 1 (2.56) | 31 (79.49) |
| 20-24 years | 2 (2.67) | 19 (25.33) | 1 (1.33) | 18 (24.00) | 4 (5.33) | 57 (76.00) |
| 25-29 years | 2 (2.11) | 31 (32.63) | 3 (3.16) | 30 (31.58) | 14 (14.74) | 67 (70.53) |
| 30-34 years | 2 (2.82) | 27 (38.03) | 1 (1.41) | 22 (30.99) | 11 (15.49) | 53 (74.65) |
| 35-39 years | 0 (0.00) | 15 (26.79) | 0 (0.00) | 16 (28.57) | 8 (14.29) | 46 (82.14) |
| 40 years and above | 1 (0.83) | 38 (31.40) | 6 (4.96) | 35 (28.93) | 23 (19.01) | 86 (71.07) |
| **Ethnicity** |  |  |  |  |  |  |
| Hill Dalit | 2 (3.45) | 20 (34.48) | 1 (1.72) | 14 (24.14) | 12 (20.69) | 43 (74.14) |
| Terai Dalit | 0 (0.00) | 12 (40.00) | 0 (0.00) | 15 (50.00) | 5 (16.67) | 21 (70.00) |
| Hill Janajati | 4 (4.21) | 33 (34.74) | 1 (1.05) | 26 (27.37) | 13 (13.68) | 71 (74.74) |
| Terai Janajati | 1 (0.88) | 34 (29.82) | 2 (1.75) | 34 (29.82) | 15 (13.16) | 80 (70.18) |
| Brahman/Chhetri | 3 (1.88) | 43 (26.88) | 8 (5.00) | 42 (26.25) | 16 (10.00) | 125 (78.12) |
| **Religion** |  |  |  |  |  |  |
| Hindu | 7 (1.87) | 114 (30.48) | 10 (2.67) | 115 (30.75) | 48 (12.83) | 277 (74.06) |
| Buddhist | 1 (3.57) | 11 (39.29) | 0 (0.00) | 5 (17.86) | 7 (25.00) | 23 (82.14) |
| Muslim | 1 (5.56) | 6 (33.33) | 2 (11.11) | 3 (16.67) | 3 (22.22) | 10 (55.56) |
| Kirat | 0 (0.00) | 2 (20.00) | 0 (0.00) | 5 (50.00) | 0 (0.00) | 10 (100.0) |
| Christian | 1 (3.70) | 9 (33.33) | 0 (0.00) | 3 (11.11) | 2 (7.41) | 20 (74.07) |
| **Education** |  |  |  |  |  |  |
| No | 4 (1.67) | 65 (27.20) | 4 (1.67) | 58 (24.27) | 27 (11.30) | 187 (78.24) |
| Yes | 6 (2.75) | 77 (35.32) | 8 (3.67) | 73 (33.49) | 34 (15.60) | 153 (70.18) |
| **Provinces** |  |  |  |  |  |  |
| Province 1 | 2 (1.60) | 28 (22.40) | 2 (1.60) | 54 (43.20) | 13 (10.40) | 117 (93.60) |
| Province 2 | 0 (0.00) | 31 (32.29) | 0 (0.00) | 45 (46.88) | 17 (17.71) | 72 (75.00) |
| Bagmati | 3 (3.33) | 23 (25.56) | 3 (3.33) | 15 (16.67) | 4 (4.44) | 62 (68.89) |
| Gandaki | 0 (0.00) | 16 (31.37) | 0 (0.00) | 7 (13.73) | 10 (19.61) | 35 (68.63) |
| Lumbini | 5 (19.23) | 11 (42.31) | 3 (11.54) | 2 (7.69) | 2 (7.69) | 4 (15.38) |
| Karnali | 0 (0.00) | 29 (51.79) | 4 (7.14) | 7 (12.50) | 9 (16.07) | 42 (75.00) |
| Sudurpaschim | 0 (0.00) | 4 (30.77) | 0 (0.00) | 1 (7.69) | 6 (46.15) | 8 (61.54) |

**Type of disability and violence experienced**

The analysis reveals that individuals with intellectual disabilities reported the highest prevalence of sexual violence (23.81%). Physical violence was most prevalent among those with hearing disabilities (55.56%), while denial of services was most commonly reported by individuals with vocal and speech-related disabilities (45.45%). Forced or child marriage was highest among those with deaf-blind disabilities (12.50%), whereas spousal violence was most prevalent among individuals with hearing disabilities (33.33%). We also found that psychological violence was more prevalent among individuals with vision-related disabilities (85.51%) (see Figure 1).

**<Insert Figure 1>**

**Factors associated with violence against women living with disabilities**

Table 3 shows the results for the bivariable and multivariable logistic regression models. In the adjusted model, age was positively associated with the likelihood of experiencing violence, with women aged 25–29 years [AOR=2.17; 95%CI: 1.29–3.65], 30–34 years [AOR=2.66; 95%CI: 1.51–4.68], and 35–39 years [AOR=2.16; 95%CI: 1.20–3.90] having significantly higher odds compared to those aged 15–19 years. Women belonging to the Brahman/Chhetri ethnic group had reduced odds of violence [AOR=0.56; 95%CI: 0.37–0.85] compared to Hill Dalits. Divorced or separated women showed a markedly higher likelihood of experiencing violence [AOR=6.69; 95%CI: 2.31–19.40] compared to currently married women. Participants who had not witnessed violence against other women exhibited significantly lower odds of experiencing violence [AOR=1.86; 95%CI: 1.20–2.89]. Women living in provinces other than Province 1 reported significantly reduced odds of experiencing violence, with the lowest odds observed in Sudurpaschim [AOR=0.04; 95%CI: 0.02–0.09] and Lumbini [AOR=0.08; 95%CI: 0.05–0.15].

**Table 3: Factors associated with violence against women living with disabilities**

| **Variables** | **Unadjusted Model**  **OR [95%CI]** | **Adjusted Model**  **AOR [95%CI]** |
| --- | --- | --- |
| **Age** |  |  |
| 15-19 years | Ref. | Ref. |
| 20-24 years | **2.03 [1.29-3.19]**** | **1.78 [1.06-2.98]*** |
| 25-29 years | **2.40 [1.55-3.72]***** | **2.17 [1.29-3.65]**** |
| 30-34 years | **2.87 [1.79-4.59]***** | **2.66 [1.51-4.68]**** |
| 35-39 years | **2.54 [1.55-4.15]***** | **2.16 [1.20-3.90]*** |
| 40 years and above | **1.92 [1.27-2.91]**** | **1.88 [1.10-3.21]*** |
| **Ethnicity** |  |  |
| Hill Dalit | Ref. | Ref. |
| Terai Dalit | **2.20 [1.22-3.97]**** | 0.91 [0.40-2.06] |
| Hill Janajati | **1.68 [1.11-2.52]*** | 0.79 [0.49-1.27] |
| Terai Janajati | **1.67 [1.13-2.48]*** | 0.89 [0.51-1.56] |
| Brahman/Chhetri | 0.93 [0.65-1.34] | **0.56 [0.37-0.85]**** |
| **Religion** |  |  |
| Hindu | Ref. | - |
| Buddhist | **3.03 [1.67-5.49]***** | - |
| Muslim | **2.46 [1.23-4.94]*** | - |
| Kirat | **10.27 [2.24-47.12]**** | - |
| Christian | 1.68 [0.99-2.84] | - |
| **Education** |  |  |
| No | Ref. | - |
| Yes | 0.97 [0.77-1.22] | - |
| **Marital status** |  |  |
| Currently married | Ref. | Ref. |
| Never married | 0.79 [0.63-1.01] | 0.89 [0.66-1.21] |
| Divorced/separated | **8.33 [3.39-20.40]***** | **6.69 [2.31-19.40]***** |
| Widowed | 0.93 [0.53-1.65] | 1.51 [0.73-3.12] |
| **Age at first marriage** |  |  |
| Before 20 years | Ref. | - |
| At 20 year or older | **1.56 [1.16-2.09]**** | - |
| **Receive disability allowance** |  |  |
| No | Ref. | - |
| Yes | 1.13 [0.89-1.42] | - |
| **Knowledge on policies and laws that support victims of violence** |  |  |
| Yes | Ref. | - |
| No | 1.26 [0.94-1.68] | - |
| **Ever witnessed violence against women** |  |  |
| Yes | Ref. | Ref. |
| No | **3.11 [2.10-4.60]***** | **1.86 [1.20-2.89]**** |
| **Provinces** |  |  |
| Province 1 | Ref. | Ref. |
| Province 2 | **0.54 [0.35-0.82]**** | **0.54 [0.29-0.98]*** |
| Bagmati | **0.44 [0.29-0.68]***** | **0.55 [0.35-0.84]**** |
| Gandaki | **0.17 [0.11-0.26]***** | **0.21 [0.13-0.33]***** |
| Lumbini | **0.08 [0.05-0.13]***** | **0.08 [0.05-0.15]***** |
| Karnali | **0.20 [0.13-0.31]***** | **0.25 [0.15-0.39]***** |
| Sudurpaschim | **0.04 [0.02-0.08]***** | **0.04 [0.02-0.09]***** |

*p<0.05, **p<0.01, ***p<0.001; Ref: Reference category

***(-)*** *Variables excluded after backward stepwise approach*

**Discussion**

This study investigated the lifetime prevalence of violence against women and girls living with disabilities in Nepal. We found that 35.32% of the participants had ever experienced violence in their lifetime. While the estimated prevalence of violence among women living with disabilities is lower than what has been previously reported in another Nepalese study (58%) [11], it resonates with the global statistics where one in three women experience violence [1]. Our findings also align with a recent systematic review of 29 LMICs that showed that women with disabilities are twice as likely to experience violence compared to their counterparts without disabilities [20]. The results also resonate with that of developed countries like Australia, and the USA [21,22]. Thus, emphasizing the immediate necessity for focused interventions and assistance programs for girls with disabilities in Nepal.

Consistent with previous studies [1,23,24], we found psychological violence to be the most prevalent violence type, followed by physical violence. The low prevalence of sexual violence observed in this study could be explained from the perspective that victims of such violence are highly stigmatized and thus, are unlikely to report their experience for fear of shame, blame, or ostracization from their families and communities [23,25]. Nonetheless, compared to other types of disabilities, sexual violence appears to be pervasive among those living with intellectual disabilities. Our observation aligns with previous literature that have found sexual violence to four times more prevalent among persons living with intellectual disabilities than any other group in the disability spectrum [26,27]. Perhaps, the reasoning challenges of women with intellectual disabilities connote a notion that they would be unable to report acts of sexual violence. As such, perpetrators of this violence take advantage of their cognitive impairments.

Women living with vocal or speech disabilities were more vulnerable to denial of services. This finding corroborates Stransky et al.’s study [28] that found that individuals with communication disabilities, including speech impairments, had a difficult time accessing healthcare services. We argue that this finding reflects the lack of inclusive service systems that accommodate alternative communication methods such as sign language and augmentative devices. It is also possible that members of the society may perceive individuals with vocal and speech-related disabilities as less capable of benefiting from certain services, leading to their exclusion from key services (e.g., sexual and reproductive health services, financial services, etc.). The elevated prevalence of forced or child marriage among the deaf-blind may be tied to societal biases perceiving them as burdens; hence, families lean towards such arrangements to reduce caregiving responsibilities.

Our study found that as the ages of women with disability increases, the likelihood of them being exposed to violence increases. This is inconsistent with a previous study that found an inverse association between age and risk of violence victimization [29]. However, the disaggregated data shows that sexual violence was more prevalent among young girls (15-19 years) than older women. Perhaps, the high prevalence of sexual violence among young girls could be attributed to lack the autonomy, knowledge, or resources for this key population to protect themselves from sexual harassments and exploitations. at the where it found that an increase in the year of women reduces their risk of facing violence by four percent. Literature also shows that young girls living with disabilities in Nepal often lack access to sexual and reproductive health information and services. As such, they are likely to be sexually empowered, and this exacerbates their vulnerabilities to sexual violence [30].

The results revealed that compared to currently married women, those who were previously married (i.e., divorced/separated) were significantly more likely to have experienced violence. This is inconsistent with previous studies [31,32] that found that married people are more exposed to violence. However, the result is corroborated by one study that shows a high prevalence of risk of violence among divorced women [33]. One plausible interpretation may be that divorced women are more likely to be subjected to ridicule, verbal abuse and in some cases, denied access to critical services as they are believed to bring bad luck [34].

Compared to Province 1, women with disabilities in all other provinces were significantly less likely to experience violence, with the likelihood of experiencing violence being 96% lower among women in Sudurpaschim. To the best of our knowledge, there are no published studies directly comparing this observation. However, a study by Kakchapati et al. [30] reported that women with disabilities in Karnali and Sudurpaschim have greater access to sexual and reproductive health services. This access may indirectly contribute to reduced vulnerability to violence by exposing women to education on violence prevention, recognition of different forms of violence, and strategies to protect themselves in such circumstances.

In contrast to earlier studies that have found a significant inverse association between education and risk of violence among women with disabilities [35,36], we found no significant association. Also, women with formal education had a higher prevalence of experiencing violence. Our finding is synonymous to Puri et al.’s study [11] that found no statistically significant association between education and risk of experiencing violence among persons living with disabilities in Nepal. One possible explanation is that formal education may increase visibility and social interactions, inadvertently exposing women with disabilities to a broader spectrum of societal discrimination and violence. Moreover, systemic factors such as stigmatization or lack of enforcement of disability rights may undermine the protective role of education.

**Implications for Policy and Practice**

Evidence from this study underscores a need for the Nepalese government to prioritize the development and enforcement of comprehensive, disability-inclusive anti-violence legislation that addresses the unique risks identified in this study. For instance, targeted interventions are essential to protect women with intellectual disabilities from sexual violence, including community education programs to challenge stigma and enforce strict penalties against perpetrators who exploit cognitive impairments. Additionally, there is an urgent need to make service delivery systems more inclusive by incorporating alternative communication methods, such as sign language and augmentative communication devices, to reduce the exclusion experienced by women with vocal and speech-related disabilities. The provincial disparities highlight a need to assess the individual provinces’ current interventions aimed at preventing violence against women living with disabilities. Specifically, Province 1 could learn some best practices from the other provinces such as Sudurpaschim.

**Strengths and limitations**

The focus of the study on PWDs is one of the strengths as it exposes a less explored area in Nepal. Also, the sample size is large enough to make extrapolations to the larger population of women and girls living with disability in Nepal. Nonetheless, the cross-sectional nature precludes us from making any sort of causal inferences. Also, as a study that follows a quantitative approach, we are unable to account for the role of cultural beliefs and normative systems in influencing the experience of violence among this vulnerable population. Also, the study does not include males and older people (i.e., 60 and over). Consequently, the findings may not be generalizable to these extended populations. Additionally, the self-reported nature of the study and the fact that participants had to recall their experience of violence may have resulted in some recall bias and social desirability biases.

**Conclusion**

In conclusion, the experience of violence varies significantly by disability type. There is a need for comprehensive, disability-inclusive policies that address the specific risks identified. Policy interventions in Nepal must focus on improving access to services for women with vocal and speech-related disabilities, ensuring that healthcare and support services are inclusive of alternative communication methods. Additionally, tailored support for divorced or separated women and age-appropriate interventions for young girls with disabilities are crucial in mitigating their increased vulnerability to violence.

**Abbreviations**

AOR: Adjusted Odds Ratio; PWDs: Persons with Disabilities; CI: Confidence Interval; NDWA: Nepal Disabled Women Association; NHRC: Nepal Health Research Council; WHO: World Health Organization

**Declaration**

**Acknowledgements**

We like to thank all women and girls who completed the survey questionnaire. We also thank staff at NDWA and Green Tara Nepal (GTN) for their support in the field and the United Nations Women Trust Fund for funding this study.

**Competing interest**

The authors have declared that no competing interests exist.

**Funding**

We received funding from the United Nations Women Trust Fund.

**Availability of data and materials**

The data for this study is available upon reasonable request from Green Tara Nepal. Requests can be directed to info@greentara.org.np. Please note that access to the data is subject to approval by Green Tara Nepal and may require ethical clearance.

**Ethical approval**

Ethical approval was provided by the Nepal Health Research Council (NHRC) (registration number 339/2020P). Both written and oral informed consent was sought from all participants. Each participant was assured of utmost confidentiality and privacy. We assured them that the data collected will be anonymised to protect their identities.

**Authors’ Contributions**

**PS**: Conceptualization, Methodology, Funding, Project Administration, Supervision, Writing - Original Draft, Writing - Review and Editing

**SB**: Conceptualization, Investigation, Methodology, Funding, Writing - Original Draft, Writing - Review and Editing

**SS**: Conceptualization, Investigation, Methodology, Funding, Writing - Original Draft, Writing - Review and Editing

**EVT**: Conceptualization, Methodology, Funding, Project Administration, Supervision, Writing - Original Draft, Writing - Review and Editing

**SPW**: Conceptualization, Methodology, Project Administration, Writing - Original Draft, Writing - Review and Editing

**TD**: Investigation, Methodology, Validation, Writing - Original Draft, Writing - Review and Editing

**JO**: Methodology, Data Curation, Formal Analysis, Software, Writing - Original Draft, Writing - Review and Editing

**RCS**: Conceptualization, Investigation, Methodology, Funding acquisition, Writing - Review and Editing

**MP**: Investigation, Methodology, Writing - Original Draft, Writing - Review and Editing

..

**References**

1. World Health Organization. *Violence against women prevalence estimates, 2018: global, regional and national prevalence estimates for intimate partner violence against women and global and regional prevalence estimates for non-partner sexual violence against women.* Geneva: World Health Organization, 2021. <https://www.who.int/publications/i/item/9789240022256>
2. World Health Organization. *Global report on health equity for persons with disabilities*. Geneva: World Health Organization, 2022.
3. National Statistics Office. National population and housing census 2021. Thapathali, Kathmandu: National Statistics Office; 2023.
4. UNICEF Nepal. An Innovative Model for Disability Screening and Issuing Disability Identity Card a Study Report 2022. <https://www.unicef.org/nepal/reports/innovative-model-disability-screening-and-issuing-disability-id-cards>.
5. Karki J, Rushton S, Bhattarai S, De Witte L. Access to assistive technology for persons with disabilities: a critical review from Nepal, India and Bangladesh. Disabil Rehabil Assist Technol, 2023;18(1): 8-16. https://doi.org/10.1080/17483107.2021.1892843
6. Mitchell D, Snyder SL. (2019). Minority model: From liberal to neoliberal futures of disability. In: Watson N, Roulstone A, Thomas C (eds).  *Routledge handbook of disability studies Second edition* (pp. 45-54). Routledge.
7. Sweileh W. Analysis and mapping of research on barriers to mental health service utilization in minority and underserved groups (1993-2022). *Mental Health Soc Inclusion* 2024. [online ahead of print] <https://doi.org/10.1108/MHSI-10-2023-0109>
8. Meyer SR, Stöckl H, Vorfeld C, Kamenov K, García-Moreno C. A scoping review of measurement of violence against women and disability. PLoS One, 2022;17(1): e0263020. <https://doi.org/10.1371/journal.pone.0263020>
9. Dembo RS, Mitra M, McKee M. The psychological consequences of violence against people with disabilities. Disability Health J 2018;11(3), 390-397. <https://doi.org/10.1016/j.dhjo.2018.01.006>
10. Iudici A, Antonello A, Turchi G. Intimate partner violence against disabled persons: Clinical and health impact, intersections, issues and intervention strategies. Sex Cult, 2019;23:684-704. https://doi.org/10.1007/s12119-018-9570-y
11. Puri M, Misra G, Hawkes S. Hidden voices: prevalence and risk factors for violence against women with disabilities in Nepal. BMC Public Health. 2015;15(1), 1-11. <https://doi.org/10.1186/s12889-015-1610-z>.
12. Thapa R, van Teijlingen E, Regmi P, Heaslip V. Caste exclusion and health discrimination in South Asia: A systematic review, Asia Pacific J Public Health 2021;33(8):828-838. <https://doi.org/10.1177/10105395211014648>
13. Colombini M, Mayhew SH, Hawkins B, Bista M, Joshi SK, Schei B, Watts C. Agenda setting and framing of gender-based violence in Nepal: how it became a health issue. Health Pol Plann 2016;31(4), 493-503. <https://doi.org/10.1093/heapol/czv091>
14. United Nations Women (2021). Nepal’s gender-based violence and gender equality funds: the path to effective implementation. policy brief. Sigma Press, Sanepa, Lalitpur. Retrieved from: <https://asiapacific.unwomen.org/en/digital-library/publications/2021/06/the-path-to-effective-implementation#view>. Accessed: January 14, 2024.
15. Olson RM, Garcia-Moreno C, Colombini M. The implementation and effectiveness of the one stop centre model for intimate partner and sexual violence in low-and middle-income countries: a systematic review of barriers and enablers. BMJ Glob Health. 2020;5(3). https://doi.org/[10.1136/bmjgh-2019-001883](https://doi.org/10.1136%2Fbmjgh-2019-001883)
16. Gupta J, Cardoso LF, Ferguson G, Shrestha B, Shrestha PN, Harris C. et al. Disability status, intimate partner violence and perceived social support among married women in three districts of the Terai region of Nepal. BMJ Global Health*.* 2018;3(5). https://doi.org/[10.1136/bmjgh-2018-000934](https://doi.org/10.1136%2Fbmjgh-2018-000934)
17. Shi F. Study on a Stratified Sampling Investigation Method for Resident Travel and the Sampling Rate, Discrete Dyn Nat Soc 2015;Article ID 496179. <https://doi.org/10.1155/2015/496179>
18. van Teijlingen E, Hundley V. Pilot studies in family planning and reproductive health care, J Family Plann Reprod Health Care 2015;31(3): 219–22.
19. Poloju KK, Naidu VR, Rollakanti CR, Manchiryal RK, Adams J. New method of data collection using the Kobo Toolbox. J Posit School Psychol. 2022; 6(4): 1527-1535.
20. Emerson E, Llewellyn G. Exposure of women with and without disabilities to violence and discrimination: evidence from cross-sectional national surveys in 29 middle-and low-income countries. J Interpers Violence, 2023;38(11-12), 7215-7241. <https://doi.org/10.1177/08862605221141868>
21. Breiding MJ, Armour BS. The association between disability and intimate partner violence in the United States. Ann Epidemiol*,* 2015;25(6), 455-457. <https://doi.org/10.1016/j.annepidem.2015.03.017>
22. Krnjacki L, Emerson E, Llewellyn G, Kavanagh AM. Prevalence and risk of violence against people with and without disabilities: Findings from an Australian population‐based study. Aust N Z J Public Health*,* 2016;40(1): 16-21. https://doi.org/10.1111/1753-6405.12498.
23. Dickson KS, Ayebeng C, Okyere J. Unveiling Shadows: Investigating women’s experience of intimate partner violence in Ghana through the lens of the 2022 Demographic and Health Survey. Plos one. 2024 Aug 29;19(8):e0309564.
24. Ringwald B, Tolhurst R, Taegtmeyer M, Digolo L, Gichuna G, Gaitho MM, Phillips–Howard PA, Otiso L, Giorgi E. Intra-urban variation of intimate partner violence against women and men in Kenya: evidence from the 2014 Kenya Demographic and Health Survey. Journal of interpersonal violence. 2023 Mar;38(5-6):5111-38.
25. Ledingham E, Wright GW, Mitra M. Sexual violence against women with disabilities: experiences with force and lifetime risk. American journal of preventive medicine. 2022 Jun 1;62(6):895-902.
26. Tomsa R, Gutu S, Cojocaru D, Gutiérrez-Bermejo B, Flores N, Jenaro C. Prevalence of sexual abuse in adults with intellectual disability: Systematic review and meta-analysis. International journal of environmental research and public health. 2021 Feb;18(4):1980.
27. Willott S, Badger W, Evans V. People with an intellectual disability: under-reporting sexual violence. The Journal of Adult Protection. 2020 Apr 21;22(2):75-86.
28. Stransky ML, Jensen KM, Morris MA. Adults with communication disabilities experience poorer health and healthcare outcomes compared to persons without communication disabilities. Journal of general internal medicine. 2018 Dec;33:2147-55.
29. Brownridge DA. Partner violence against women with disabilities: Prevalence, risk, and explanations. Violence Against Women, 2006;12(9):805-822. <https://doi.org/10.1177/1077801206292681>.
30. Kakchapati S, Kc SP, Giri S, Bhattarai S, Baral SC. Factors associated with access to sexual and reproductive health services among women with disabilities in Nepal. Dialogues in Health. 2022 Dec 1;1:100068.
31. Meseguer-Santamaría M L, Sánchez-Alberola F, Vargas-Vargas M. Intimate partner violence against women with disabilities in Spain: A public health problem. Int J Environ Res Public Health*,* 2021;18(2), 728. <https://doi.org/10.3390/ijerph18020728>
32. Sasseville N, Maurice P, Montminy L, Hassan G, St-Pierre É. Cumulative contexts of vulnerability to intimate partner violence among women with disabilities, elderly women, and immigrant women: Prevalence, risk factors, explanatory theories, and prevention. Trauma, Violence Abuse. 2022;23(1): 88-100. <https://doi.org/10.1177/1524838020925773>
33. Adak N, Elmas Ç, Kuşoğlu GC. Does marital status affect violence against women? A perusal on Turkey. Süleyman Demirel Üniversitesi Fen-Edebiyat Fakültesi Sosyal Bilimler Dergisi. 2021(54):149-64.
34. DeKeseredy WS, Dragiewicz M, Schwartz MD. Abusive endings: Separation and divorce violence against women. Univ of California Press; 2017 May 16.
35. Kouanda S, Arelatan J, Ouedraogo HG, Millogo T, Bagnoa C, Busière S, Sondo B. Prevalence and risk factors of violence among persons with disabilities in Burkina Faso. AIDS care. 2022 Jul 25;34(sup1):18-23.
36. Gebrekirstos LG, Lencha TT, Asgedom YS, Wube TB. Prevalence and factors associated with violence; perceived social support and health access among people with disabilities in rural districts of southern Ethiopia: mixed study. Journal of Multidisciplinary Healthcare. 2023 Dec 31:2921-36.
