# Supplementary material for: Life-time experience of violence among women and girls living with disability in Nepal
Source: PLoS One. 2025 Jun 25;20(6):e0326659. doi: 10.1371/journal.pone.0326659 (PMC12193803; doi:10.1371/journal.pone.0326659)
Supplement: Attachment — Submitted filename: Rebuttal letter+EvT.docx [file pone.0326659.s003.docx]

**Reply to the editor and reviewers**

Dear Editor,

Thank you for the reviewer’ comments, we have outlined our replies for each comment below;

| **Comments** | **Response** |
| --- | --- |
| **Abstract** |  |
| “A total of 1,294 questionnaires were completed…” Rewrite it as ‘A total of 1294 participants were asked…’ | Thank you for the comment, now revised: “A total of 1,294 women and girls with disability aged 15 to 59 years participated in this study.” |
| “Cross -tabulations and chi-square tests were performed in STATA 18”. Please add, how did you collect data and who collected and storage with privacy. Then revise your data analysis technique. | We have now addressed this: “Data was collected between August 30, 2021 to October 29, 2021. This was done by trained enumerators using the KoBo application on smartphones or tablets” |
| “bivariable and multivariable logistic regression models were fitted to establish associations”. Association between what? | Thank you for question, we now revised this to: “bivariable and multivariable logistic regression models were fitted to establish association between the participants’ characteristics and odds of experiencing violence” |
| Please add, how did you collect data and who collected and storage with privacy. Then revise your data analysis technique. | We have now included this in the abstract: “Data was collected between August 30 and October 29, 2021. This was done by trained enumerators using the KoBo application on smartphones or tablets. Both written and oral informed consent was sought from all participants. Each participant was assured of utmost confidentiality and privacy.” |
| Keywords should be arranged in alphabetic order | This change has been done. Thank you. |
| Please rewrite conclusion with the main findings and then lastly recommendation based on your results | This change has been made: “The study concludes that age, ethnicity, marital status, and provincial residence are significant predictors of violence among women living with disability in Nepal. Interventions aimed at addressing violence against women living with disability in Nepal must prioritize older women and those who were previously married. Also, priority must be given to Koshi where the prevalence and risk of experiencing violence is highest.” |
| **Background** |  |
| Change background to introduction | This change has been made. |
| Add little about what factors affected lifetime violence, e.g. country’s crises or any pandemic like COVID-19 cause domestic violence. Please add this paper as reference <https://jkahs.org.np/jkahs/index.php/jkahs/article/view/552/420>. | This study has been cited here: “In the general population of women, the violence against women in Nepal was exacerbated by the COVID-19 pandemic [7]. ” |
| **Methods** |  |
| This paper was written using secondary source of data. So, You have to clearly write data course under methods then go to study population and design, sampling techniques was also done during DHS and MICS but not by you. Write clearly what you have done using data collected by others. | Thank you for the comment. However, we have to make this clarification. This study is a primary study. Data was collected by the team. We only adopted questions from the DHS and MICS. We apologize for the confusion in our initial submission. |
| We conducted quantitative surveys in 14 districts representing all seven of Nepal’s provinces (Province 1, Province 2, Bagmati, Gandaki, Lumbini, Karnali, and Sudurpaschim).  Please write correct name of provinces | We have now addressed this: “Koshi, Madhesh, Bagmati, Gandaki , Lumbini, Karnali, and Sudurpaschim”  This was not a mistake in original submission at the time provices 1+2 did not have an official name yet. |
| **Results** |  |
| I do not think you need to keep this information to present prevalence. I suggest you to keep frequency and percentage only. | Thank you for suggestion. However, having this here adds more information. Here p-values show the significant differences in the distribution of the estimated prevalence. We have removed chi-square values but left the p-values. |
| “Brahman /Chhetri”  Please consider correct spelling in consistent way | We used a consistent spelling. You can see others: <https://www.bing.com/ck/a?!&&p=6b3f6b94f256e137cc3a0c5e8170cdffd72cc0cfe7bc1af19e053f60ca351cf5JmltdHM9MTczOTE0NTYwMA&ptn=3&ver=2&hsh=4&fclid=2ff5e4b0-faed-63f1-2194-f7a4fbf06259&psq=Brahman+%2fChhetri&u=a1aHR0cHM6Ly93d3cuZXZlcnljdWx0dXJlLmNvbS9Tb3V0aC1Bc2lhL0JyYWhtYW4tYW5kLUNoaGV0cmktb2YtTmVwYWwtTWFycmlhZ2UtYW5kLUZhbWlseS5odG1s&ntb=1>; <https://www.bing.com/ck/a?!&&p=51cac4a58616754b88d71a30cddcdd0328d57a5eab1aaa5226d369f07a3ea182JmltdHM9MTczOTE0NTYwMA&ptn=3&ver=2&hsh=4&fclid=2ff5e4b0-faed-63f1-2194-f7a4fbf06259&psq=Brahman+%2fChhetri&u=a1aHR0cHM6Ly9mYWN0c2FuZGRldGFpbHMuY29tL3NvdXRoLWFzaWEvTmVwYWwvTGlmZV9OZXBhbC9lbnRyeS03ODQ0Lmh0bWw&ntb=1>; |
| What is your education level classification? You have use national education standard and its classification. | This study looked at whether the individual had received formal education or not. |
| “**Life-time prevalence of violence among women and girls living with disabilities**” This info must be written before Table 1. Please write key information only but not all details from table | This change has been made. Thank you. |
| “We found that 35.32% of the participants had ever experienced violence in their lifetime.” Do not use pronouns in the discussion | This change has been made: “The results show that 35.32% of the participants had ever experienced violence in their lifetime.” |
