# Supplementary material for: Life-time experience of violence among women and girls living with disability in Nepal
Source: PLoS One. 2025 Jun 25;20(6):e0326659. doi: 10.1371/journal.pone.0326659 (PMC12193803; doi:10.1371/journal.pone.0326659)
Supplement: Attachment — Submitted filename: Referee report - PLOS ONE.docx [file pone.0326659.s005.docx]

Referee report: Life-time experience of violence among women and girls living with disability in Nepal: A cross-sectional study

The authors conduct an important descriptive study of the life-time experience of violence among women and girls living with disability in Nepal, shedding light on the prevalence rates for different demographics and regions in a national survey sample and on the predictive factors associated with experiencing violence among this vulnerable population. Overall, the study is presented in an intelligible fashion and analyses are described well. My review has highlighted a few issues that – while major, as in, critical to meet publication standards – the authors should be able to address easily.

Response: Thank you for the kind words, and for the time spent to review the revised manuscript. We appreciate the constructive feedbacks.

These major issues include, in order of appearance:

- The result “Participants who had not witnessed violence against other women exhibited significantly lower odds of experiencing violence” is at odds with the AOR reported in Table 2 – these women appear to have *higher* odds of experiencing violence. This result is mentioned in the Abstract as well as Results sections.

Response: Thank you for drawing our attention. This error has been rectified. It reads: “Participants who had not witnessed violence against other women exhibited significantly higher odds of experiencing violence”.

- It would be helpful for context to understand a bit more what being “part of a larger project in Nepal to reduce violence against women with disabilities” means for the survey. Does this mean that the survey was longer than the questions presented here?

What does “project interventions” refer to, which are mentioned as a reason for an estimated 5% reduction in violence against women with disability? Does this mean that this survey comes after (experimental/government/NGO) interventions were run to reduce violence? Or is this survey the “baseline” for the larger project?

Response: Thank you for the comment. This study is part of a project that is investigating the wellbeing of women and girls living in Nepal. It covers health and social issues including violence experience. We have now indicated this: “It must be noted that this study is a baseline survey of a larger project in Nepal exploring multiple social and health dimensions of women and girls, including those living with disabilities”.

- I would like to know/see some explanation in the manuscript to whether the question about life-time experience of any form of violence was accompanied by any sort of explanation about what constitutes violence. If not, I think it is a limitation that some

girls and women may not have recognized that they were subject to violence (e.g., denial of services, psychological), and so may have answered ‘no’ to the general question and were subsequently not asked for any further detail of what specific violence type they may have experienced. In that case, the prevalence could potentially be higher and also the relative occurrence of different forms of violence could be skewed. If this is the case, this should be acknowledged in the limitations.

Response: Thank you for this insightful comment. The question did not come with any other explanations. As such, we have acknowledged this in the limitations section. It reads: “Another limitation of this study lies in how life-time experience of violence was measured. There was no prompting in the questioning. As such, it is possible that some participants may not recognize some actions (e.g., denial of service) as violence. Therefore, it is possible that the prevalence may be underestimated in this study.”

- Table 1 and associated text:
  - I would suggest being careful interpreting significant differences where groups are very small, i.e., the Kirat group with just over 20 observations in total.

Response: Thank you for the caution. However, as you may have realized, the distribution was based on row % rather than column %. That approach addresses the dangers that can potentially arise when interpreting the significant differences as you pointed out.

- The numbers in ‘Age at first marriage’ are strange; should the total for whom this is available not be equal to 550 (Currently married)+35 (Divorced)+57

(Widowed), so, excluding the 652 who were ‘never married’? If data is available for the full sample, it is unclear what the variable actually means.

Response: Thank you for drawing our attention. This correction has been made. Please see the table here.

| **Age at first marriage** |  |  | **0.002** |
| --- | --- | --- | --- |
| Never married | 652 (50.39) | 206 (31.60) |  |
| Before 20 years | 559 (46.29) | 228 (38.06) |  |
| At 20 year or older | 43 (3.32) | 23 (53.49) |  |

- Minor comment: There must be a typo in the percentage of “No” for “Knowledge on policies and laws that support victims of violence” – it cannot be 100%.

Response: Yes. That is quite observant of you. We have addressed this. Thank you: “1025 (79.21)”

- Table 2 and associated text:
  - I would make clear that this was a multi-select question that was asked of the 457 women and girls who had experienced any violence. That would then help to understand why the percentages add up to more than 100%, and what the total N is.

Response: Thank you. We have now added a footnote this this: “NB: This was a multi-select question answered by 457 participants who had every experienced any violence”

- As above, I would refrain from interpreting types of violence with very low sample, i.e., sexual violence (n=10) and forced/child marriage (n=12) (At least add the caveat that percentages in small samples are not reliable, as one single observation can change the percentage dramatically.)

Response: Thank you for the suggestion. In the revised manuscript, we have refrained from interpreting the results with a low sample.

- Type of disability and violence experienced: I think this is an interesting and important section, and it would deserve a bit more insight. As a reader, I am wondering what the distribution is of different types of disability in the sample. How common is each type of disability? And how many girls/women have more than one disability type? My

suggestion would be to add these variables to Table 1.

Response: We have now included this in Table 1 as suggested: “Specifically, 42.08% of women with a visual disability reported experiencing violence, compared to 33.62% of women without a visual disability. Similarly, 37.65% of women with a physical disability experienced violence, compared to 33.33% among those without. However, for self-care disability, the prevalence of violence was similar between women with the disability (33.92%) and those without (35.95%). In contrast, lower prevalence of violence was observed among women with hearing (23.66% vs. 36.22%), cognitive (24.46% vs. 36.62%), and communication disabilities (19.05% vs. 37.40%) compared to their counterparts without these disabilities.”

- Table 3 and associated text:
  - Please explain the backward stepwise approach that led to exclusion of certain variables. This is not mentioned anywhere in the methods or table notes.

Response: We have now included a description of this in the methods section: “In the multivariable analysis, we employed a backward stepwise logistic regression approach to build the final adjusted model. Initially, all variables with theoretical relevance and those significant at p<0.20 in the bivariate analysis were included in the full model. Variables were then sequentially removed based on the likelihood ratio test (LRT) and Akaike Information Criterion (AIC) values, ensuring that removal did not meaningfully alter the effect estimates of key predictors or degrade the model's fit. Variables with p-values above 0.05 that did not contribute significantly to model fit and lacked theoretical justification were excluded. Specifically, religion, education, age at first marriage, disability allowance receipt, and knowledge on policies and laws supporting victims were excluded in the final model due to lack of statistical significance and negligible impact on the effect estimates of retained variables”

- As a robustness test, I would suggest using a different province to Koshi Province as reference category. The reason why is that you focus a lot on Koshi Province (rightly so because it clearly has the highest prevalence, and the highest

adjusted odds), but Table 1 shows that Madhesh and Bagmati Province also have high prevalence rates (around 50%), much higher than the remaining provinces and much higher than the sample average. The fact that the odds for provinces do not change a lot when adjusted for other variables indicates to me that you might be missing some important recommendations for other provinces, that also have quite high prevalence, but show up as ‘low odds’ simply because they are compared to Koshi Province. I would choose Karnali Province (= the Province with the prevalence rate most closely aligned with the whole sample average) as reference category in the logistic regressions.

Response: Thank you for the different perspective. We have now done this: “Women living in the Koshi province [AOR=4.04; 95%CI: 2.54-6.42], Madhesh province [AOR=2.16; 95%CI: 1.15-4.08] and Bagmati province [AOR=2.21; 95%CI: 1.41-3.46] reported significantly higher odds of experiencing violence compared to those in Karnali.”

- When you discuss the association of experience of violence with age, your conclusion that this is inconsistent with reference [29] is misguided. That paper examines experience of violence in the last 5 years, not lifetime. In your setup, life-time exposure to violence increases necessarily with age, *by design* (= theoretically, it can never go down, only up or stay the same), and it says nothing about which age groups are more likely to being exposed to violence. Please avoid making such statements. Similarly, when discussing a higher prevalence of sexual violence among young girls, ‘lack of autonomy’ would have applied to older women equally when they were young. So, the fact that they haven’t reported sexual violence indicates either recall bias or that “the times are changing”, perhaps that sexual violence is much more of an issue nowadays than it was 30-50 years ago.

Response: We have revised this section accordingly: “The difference between our findings and that of Brownridge [29] stems from the point that unlike the cited study that focused on experience of violence in the last five years, our study focused on life-time experience. And so, our finding reflects the cumulative nature of lifetime exposure, where the probability of ever experiencing violence increases with age due to a longer period of risk exposure. Therefore, this association should be interpreted cautiously, as it does not imply that older women are currently at higher risk of violence victimization compared to younger women, but rather reflects accumulated experiences over the life course.”

Minor issues include:

- A few typos throughout the manuscript. Please review carefully. There is also an incomplete sentence in the fourth paragraph of the Discussion (“at the where it found that….”).

Response: We have now addressed the typos in the manuscript. Thank you.

- Reference 18 (pilot studies) lists an incorrect year of publication (2015 instead of 2005)

Response: We have now addressed this: “van Teijlingen E, Hundley V. Pilot studies in family planning and reproductive health care. BMJ Sexual & Reproductive Health. 2005 Jul 1;31(3):219.”

**Reviewer #4: Simkhada et al. Life-time experience of violence among women and girls living with disability in Nepal: A cross-sectional study**

This is a straightforward study reporting life-time prevalence of violence among women and girls living with disabilities. However, there is one major issue with the paper, that needs to be rectified.
Response: Thank you for the time spent to review the revised manuscript, and for the constructive feedbacks.

The article lacks a table on distribution by types of disabilities. The description on how disability was identified is poorly explained. Authors should clearly describe if some method was used to confirm disability, eg disability pension books, or disability card (if available).
Response: Thank you. We have now included a description of how disability was measured: “Disabilities were assessed across six functional domains: seeing, hearing, walking/climbing steps, remembering/concentrating, self-care, and communication. For each domain, participants were asked about the level of difficulty experienced, with responses categorized as: no difficulty, some difficulty, a lot of difficulty, or cannot do at all. Following the Washington Group recommendations, a binary variable was created for each domain, coded as 1 if the respondent reported “a lot of difficulty” or “cannot do at all,” and 0 otherwise. Subsequently, a composite variable was generated to identify individuals with two or more disabilities by summing the six domain-specific binary variables and recoding the total as 1 if the individual had disabilities in two or more domains, and 0 otherwise.”

The authors have mentioned that they had excluded severe intellectual disability, but elsewhere the paper states that women and girls with intellectual disabilities were at a higher risk of sexual violence.
Response: Thank you for the comment. However, there is no contradiction in the manuscript. As stated, women and girls with severe intellectual disabilities were excluded due to the lack of trained mental health interviewers capable of supporting their specialized needs during data collection. The analyses presented in the paper refer to women and girls with mild to moderate cognitive disabilities (as measured by self-reported difficulty in remembering or concentrating). Therefore, references to an increased risk of violence pertain to this group, not to those with severe intellectual impairments who were excluded from the study.

The authors should note that without clarification on how disability was measured, and the distribution of disability by type, the section on “type of disability and violence experienced” is not meaningful. The authors should incorporate these statistics in appropriate sections of the manuscript.

Response: Thank you for the comment. This detail has been included as suggested.
