# Supplementary material for: Life-time experience of violence among women and girls living with disability in Nepal
Source: PLoS One. 2025 Jun 25;20(6):e0326659. doi: 10.1371/journal.pone.0326659 (PMC12193803; doi:10.1371/journal.pone.0326659)
Supplement: Attachment — Submitted filename: Referee report - PLOS ONE.pdf [file pone.0326659.s004.pdf]

Referee report: Life-time experience of violence among women and girls living with disability in Nepal: A cross-sectional study

The authors conduct an important descriptive study of the life-time experience of violence among women and girls living with disability in Nepal, shedding light on the prevalence rates for different demographics and regions in a national survey sample and on the predictive factors associated with experiencing violence among this vulnerable population.

Overall, the study is presented in an intelligible fashion and analyses are described well. My review has highlighted a few issues that – while major, as in, critical to meet publication standards – the authors should be able to address easily.

These major issues include, in order of appearance:

- The result "Participants who had not witnessed violence against other women exhibited significantly lower odds of experiencing violence" is at odds with the AOR reported in Table 2 – these women appear to have *higher* odds of experiencing violence. This result is mentioned in the Abstract as well as Results sections.
- It would be helpful for context to understand a bit more what being "part of a larger project in Nepal to reduce violence against women with disabilities" means for the survey. Does this mean that the survey was longer than the questions presented here? What does "project interventions" refer to, which are mentioned as a reason for an estimated 5% reduction in violence against women with disability? Does this mean that this survey comes after (experimental/government/NGO) interventions were run to reduce violence? Or is this survey the "baseline" for the larger project?
- I would like to know/see some explanation in the manuscript to whether the question about life-time experience of any form of violence was accompanied by any sort of explanation about what constitutes violence. If not, I think it is a limitation that some girls and women may not have recognized that they were subject to violence (e.g., denial of services, psychological), and so may have answered 'no' to the general question and were subsequently not asked for any further detail of what specific violence type they may have experienced. In that case, the prevalence could potentially be higher and also the relative occurrence of different forms of violence could be skewed. If this is the case, this should be acknowledged in the limitations.
- Table 1 and associated text:
    - I would suggest being careful interpreting significant differences where groups are very small, i.e., the Kirat group with just over 20 observations in total.
    - The numbers in 'Age at first marriage' are strange; should the total for whom this is available not be equal to 550 (Currently married)+35 (Divorced)+57 (Widowed), so, excluding the 652 who were 'never married'? If data is available for the full sample, it is unclear what the variable actually means.
    - Minor comment: There must be a typo in the percentage of "No" for "Knowledge on policies and laws that support victims of violence" – it cannot be 100%.
- Table 2 and associated text:
    - I would make clear that this was a multi-select question that was asked of the 457 women and girls who had experienced any violence. That would then help to understand why the percentages add up to more than 100%, and what the total N is.

- As above, I would refrain from interpreting types of violence with very low sample, i.e., sexual violence (n=10) and forced/child marriage (n=12) (At least add the caveat that percentages in small samples are not reliable, as one single observation can change the percentage dramatically.)

- Type of disability and violence experienced: I think this is an interesting and important section, and it would deserve a bit more insight. As a reader, I am wondering what the distribution is of different types of disability in the sample. How common is each type of disability? And how many girls/women have more than one disability type? My suggestion would be to add these variables to Table 1.
- Table 3 and associated text:
    - Please explain the backward stepwise approach that led to exclusion of certain variables. This is not mentioned anywhere in the methods or table notes.
    - As a robustness test, I would suggest using a different province to Koshi Province as reference category. The reason why is that you focus a lot on Koshi Province (rightly so because it clearly has the highest prevalence, and the highest adjusted odds), but Table 1 shows that Madhesh and Bagmati Province also have high prevalence rates (around 50%), much higher than the remaining provinces and much higher than the sample average. The fact that the odds for provinces do not change a lot when adjusted for other variables indicates to me that you might be missing some important recommendations for other provinces, that also have quite high prevalence, but show up as ‘low odds’ simply because they are compared to Koshi Province. I would choose Karnali Province (= the Province with the prevalence rate most closely aligned with the whole sample average) as reference category in the logistic regressions.
- When you discuss the association of experience of violence with age, your conclusion that this is inconsistent with reference [29] is misguided. That paper examines experience of violence in the last 5 years, not lifetime. In your setup, life-time exposure to violence increases necessarily with age, *by design* (= theoretically, it can never go down, only up or stay the same), and it says nothing about which age groups are more likely to being exposed to violence. Please avoid making such statements. Similarly, when discussing a higher prevalence of sexual violence among young girls, ‘lack of autonomy’ would have applied to older women equally when they were young. So, the fact that they haven’t reported sexual violence indicates either recall bias or that “the times are changing”, perhaps that sexual violence is much more of an issue nowadays than it was 30-50 years ago.

Minor issues include:

- A few typos throughout the manuscript. Please review carefully. There is also an incomplete sentence in the fourth paragraph of the Discussion (“at the where it found that....”).
- Reference 18 (pilot studies) lists an incorrect year of publication (2015 instead of 2005)
